# Supplementary material for: Causal Relationship between Aging and Anorexia Nervosa: A White-Matter-Microstructure-Mediated Mendelian Randomization Analysis
Source: Biomedicines. 2024 Aug 16;12(8):1874. doi: 10.3390/biomedicines12081874 (PMC11351342; doi:10.3390/biomedicines12081874)
Supplement: Supplementary file 1 [file biomedicines-12-01874-s001.zip › Supplementary Figure/Figure S4/Singe SNP plot of MR analysis for TL to ubm-b-1478.pdf]

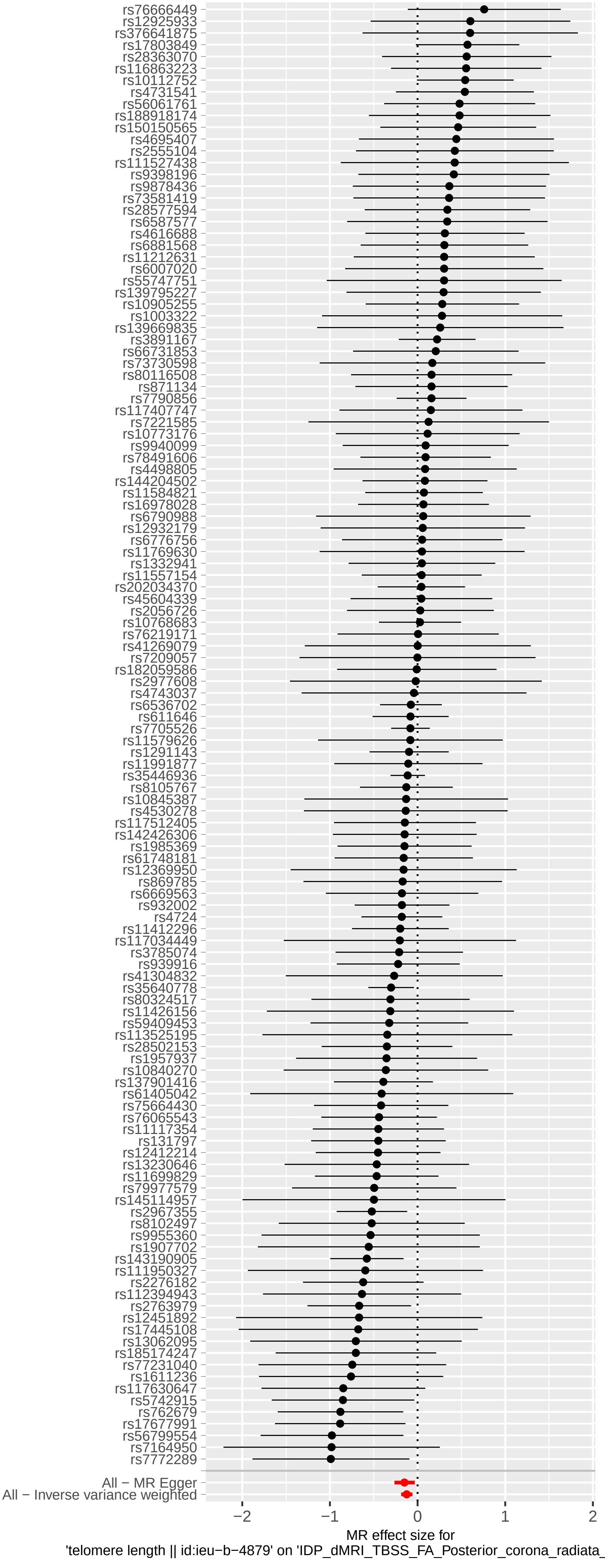
rs76666449
rs12925933
rs376641875
rs17803849
rs28363070
rs116863223
rs10112752
rs4731541
rs56061761
rs188918174
rs150150565
rs4695407
rs2555104
rs111527438
rs9398196
rs9878436
rs73581419
rs28577594
rs6587577
rs4616688
rs6881568
rs11212631
rs6007020
rs55747751
rs139795227
rs10905255
rs1003322
rs139669835
rs3891167
rs66731853
rs73730598
rs80116508
rs871134
rs7790856
rs117407747
rs7221585
rs10773176
rs9940099
rs78491606
rs4498805
rs144204502
rs11584821
rs16978028
rs6790988
rs12932179
rs6776756
rs11769630
rs1332941
rs11557154
rs202034370
rs45604339
rs2056726
rs10768683
rs76219171
rs41269079
rs7209057
rs182059586
rs2977608
rs4743037
rs6536702
rs611646
rs7705526
rs11579626
rs1291143
rs11991877
rs35446936
rs8105767
rs10845387
rs4530278
rs117512405
rs142426306
rs1985369
rs61748181
rs12369950
rs869785
rs6669563
rs932002
rs4724
rs11412296
rs117034449
rs3785074
rs939916
rs41304832
rs35640778
rs80324517
rs11426156
rs59409453
rs113525195
rs28502153
rs1957937
rs10840270
rs137901416
rs61405042
rs75664430
rs76065543
rs11117354
rs131797
rs12412214
rs13230646
rs11699829
rs79977579
rs145114957
rs2967355
rs8102497
rs9955360
rs1907702
rs143190905
rs111950327
rs2276182
rs112394943
rs2763979
rs12451892
rs17445108
rs13062095
rs185174247
rs77231040
rs1611236
rs117630647
rs5742915
rs762679
rs17677991
rs56799554
rs7164950
rs7772289
All − MR Egger
All − Inverse variance weighted
−2
−1
0
1
2
MR effect size for
'telomere length || id:ieu−b−4879' on 'IDP_dMRI_TBSS_FA_Posterior_corona_radiata_
